# Depressive symptoms and apathy are associated with psychomotor slowness and frontal activation

**DOI:** 10.1007/s00406-012-0296-9

**Published:** 2012-02-10

**Authors:** Masayo Sawa, Hidehisa Yamashita, Koichiro Fujimaki, Go Okada, Terumichi Takahashi, Shigeto Yamawaki

**Affiliations:** 1 Daijikai Mihara Hospital, 6-31-1 Nakano-cho, Mihara, Hiroshima, 723-0003 Japan; 2 Department of Psychiatry and Neurosciences, Graduate School of Biomedical Sciences, Hiroshima University, 1-2-3 Kasumi, Minami-ku, Hiroshima, 734-8551 Japan; 3 Department of Occupational Therapy, Faculty of Health and Welfare, Prefectural University of Hiroshima, 1-1 Gakuen-cho, Mihara, Hiroshima, 723-0053 Japan; 4 Department of Psychiatry, University of Michigan, 4250 Plymouth Road, Ann Arbor, MI 8109-2700 USA

**Keywords:** Depressive symptoms, Apathy, Psychomotor slowness, Cortical activation, Quality of life

## Abstract

Affective symptoms, such as depression and apathy, and cognitive dysfunction, such as psychomotor slowness, are known to have negative impacts on the quality of life (QOL) of patients with mental and physical diseases. However, the relationships among depressive symptoms, apathy, psychomotor slowness, and QOL in a non-clinical population are unclear. The aim of the present study was to assess these relationships and examine the underlying cortical mechanisms in a non-clinical population. Fifty-two healthy male volunteers were assessed for depressive symptoms using the Zung Self-rating Depression Scale (SDS), for apathy measured using the Apathy Scale, and QOL using the Short-Form 36 item questionnaire (SF36). The volunteers also performed the Trail Making Test Part A (TMT-A) while undergoing assessment of hemoglobin concentration changes in the frontal cortical surface using 24-channel near-infrared spectroscopy (NIRS). The scores of the SDS and Apathy Scale showed significant negative correlations with the scores of most of subscales of the SF36. In addition, the SDS score had a significant positive correlation with the time to complete the TMT-A. Further, activation of several frontal cortical areas had a significant positive correlation with the scores of the SDS and Apathy Scale. These results suggest that the degree of depressive symptoms and apathy are associated with a lower QOL in a non-clinical population and that cortical hyperactivation during a psychomotor task measured by NIRS may identify objectively individuals with a high degree of depressive symptoms and apathy.

## Introduction

Depressive symptoms and apathy have major impacts on the mental and physical health of individuals. Major depressive disorder (MDD), for example, is characterized by depressive symptoms and loss of interest, which is a component of apathy, and is a leading cause of worldwide disability. Worsening of depressive symptoms is associated with a reduced quality of life (QOL) [7, 17, 31]. The presence of subsyndromal depressive symptoms has also been shown to have a negative impact on psychosocial functioning [9]. In addition, there is increasing evidence that depressive symptoms are influential in the onset or progression of various kinds of diseases including Alzheimer’s disease [25], coronary disease [11], and diabetes [1]. Furthermore, there is substantial evidence suggesting the negative impacts of depressive symptoms and apathy on QOL in many diseases including HIV [33], Parkinson’s disease [21, 29], and brain tumors [14].

In addition to depressive symptoms and apathy, cognitive decline such as psychomotor slowness has a negative impact on social functioning of individuals. For example, Naismith et al. [20] reported that objectively measured psychomotor slowness is a significant predictor of physical disability in MDD, and Muslimovic et al. [19] reported that psychomotor slowness has a negative effect on QOL in Parkinson’s disease. The Trail Making Test (TMT) is a popular neuropsychological instrument and is presumed to be a test of psychomotor skills [12, 27]. Functional neuroimaging studies have reported the involvement of the frontal cortical network in TMT [10, 18, 30, 40].

Meanwhile, neurocircuit abnormalities, an underlying condition in depressive symptoms and apathy in MDD, have been studied using neuroimaging approaches. For example, previous studies reported that anhedonic symptoms and depression severity were associated with reduced caudate volume [26] and decreased activation in the subgenual anterior cingulate cortex [16]. In addition, there is substantial evidence suggesting that psychomotor slowness in MDD is related to the fronto-striatal circuitry. Several studies using positron emission tomography (PET) reported that MDD patients with affective flattening and psychomotor slowness had decreased presynaptic dopamine function in the left caudate [2, 15].

In contrast to overt psychopathology such as MDD, there have been few studies that have examined the relationship among depressive symptoms, apathy and psychomotor slowness in a non-clinical population, and the cortical mechanisms of such symptomatology are unclear. Recently, the development of near-infrared spectroscopy (NIRS) has enabled non-invasive measurement of cortical activation under natural conditions, which enables examination while the subject performs a task related to psychomotor slowness such as the TMT-A. We hypothesized that the degree of depressive symptoms and apathy are associated with psychomotor slowness, as measured by TMT-A, and abnormal cortical activation, as measured by NIRS, as well as low QOL in a non-clinical population. We performed the following study to test this hypothesis directly.

## Methods

### Subjects

Fifty-two healthy male volunteers participated in this study (mean age, 37.4 ± 11.1 years). All subjects were determined to be right-handed using the Edinburgh Handedness Inventory scale [24]. Two experienced psychiatrists together excluded a participant with psychiatric symptoms above the threshold level. No subject had a history of major psychiatric disorder including major depressive disorder and anxiety disorder, neurological disorder, substance abuse, head injury, or major physical illness or was using any psychotropic medications at the time of the study. This study was approved by the Institutional Review Board of Mihara Hospital and the Prefectural University of Hiroshima. Written informed consent was obtained from each subject prior to the study.

### Assessment of depressive symptoms, apathy, and QOL

Each subject was assessed for subjective depressive symptoms, extent of apathy, and QOL.

Subjective depressive symptoms were measured using the Zung Self-rating Depression Scale (SDS), a self-rating scale that consists of 20 questionnaires. The score of the SDS ranges from 20 (best) to 80 (worst), and the average is 35.1 ± 8.0 (mean ± SD) in the Japanese normal control population [5]. A higher score of the SDS is an indicative of a relatively greater degree of depressive symptoms.

Extent of apathy was measured using the Apathy Scale, a self-rating scale for assessing a tendency of apathy that consists of 16 questionnaires. The score of the Apathy Scale ranges from 0 (best) to 42 (worst), and the average is 8.7 ± 6.6 (mean ± SD) in the Japanese normal control population [23]. A higher score of the scale is an indicative of a relatively greater degree of apathy.

QOL was measured using the Medical Outcomes Study Short-Form 36-item questionnaire (SF36) [39]. SF36 is used widely to assess physical and mental well-being in social and individual contexts. Eight subscales are derived, referring to 8 health concepts: physical functioning (SF36-PF), role functioning-physical (SF36-RP), bodily pain (SF36-BP), general health (SF36-GH), vitality (SF36-VT), social functioning (SF36-SF), role functioning-emotional (SF36-RE), and mental health (SF36-MH). Each subscale ranges from 0 (worst health) to 100 (best health), and a score of 50 represents the mean score for the population.

### Activation task

The activation task consisted of a 30-s pre-task baseline, a TMT-A, and a 70-s post-task baseline. Each subject sat on a comfortable chair in a quiet room, and the subject was ordered to keep their head immobile as much as possible and to not speak. During the test, the subjects were required to draw a line as rapidly as possible joining consecutive numbers (1–25), which were pseudorandomly arranged on each page. We used series of 4 TMT-A sheets, which had different circle position patterns. The time required for completing the test (TMT time) was determined as a measure of task performance. During the pre-task and post-task periods, the subjects were instructed to draw lines repeatedly between two spots on a paper.

### NIRS measurement

**Fig. 1:**
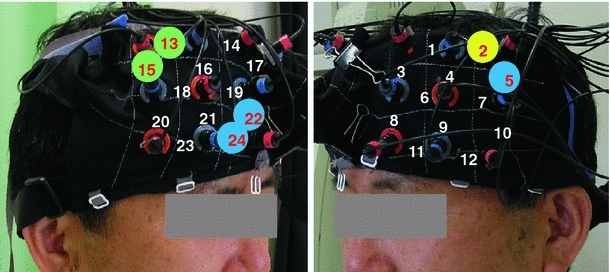
Probe setting and channels showing significant correlations with the SDS and Apathy Scale. *Yellow area* indicates a channel showing significant correlations with the SDS. *Blue areas* indicate channels showing significant correlations with the Apathy Scale. *Green areas* indicate channels showing significant correlations with both the SDS and Apathy Scale

In this study, changes in [oxy-Hb] and [deoxy-Hb] were measured using a 24-channel NIRS machine (Hitachi ETG-100) at two wavelengths of near-infrared light (i.e., 780 and 830 nm). Absorption was measured, and [oxy-Hb] and [deoxy-Hb] were calculated. The distance between the pair of emission and detector probes was 3.0 cm, and it was considered that the machine could measure points at a depth of 2–3 cm from the scalp, that is, the surface of the cerebral cortex [8, 35]. As shown in Fig. 1, the probes of the NIRS machine were placed on the subject’s bilateral frontal region. The frontal probes measured hemoglobin concentration changes at 24 measurement points in a 6 ± 15 cm area, with the lowest probes positioned along the Fp1–Fp2 line according to the international 10/20 system used in electroencephalography. The absorption of near-infrared light was measured with a time resolution of 0.1 s. The obtained data were analyzed using the ‘‘integral mode’’. The pre-task baseline was determined as the mean across the last 10 s of the 30-s pre-task period, and the post-task baseline was determined as the mean across the last 5 s of the 70-s post-task period. Linear fitting was applied to the data between these two baselines. The moving average method was used to exclude short-term motion artifacts in the analyzed data (moving average window: 5 s).

### Data analyses

The analysis focused on [oxy-Hb] changes. Changes in [oxy-Hb] were assumed to more directly reflect cognitive activation than [deoxy-Hb] changes, as shown by a stronger correlation with blood-oxygenation level-dependent (BOLD) signals measured by fMRI [32].

NIRS data that clearly contained motion artifacts determined by a close observation of the subjects were excluded from analyses.

To examine the relationship among affective symptoms (SDS, Apathy Scale) and QOL (SF36), task performances (TMT time) and [oxyHb] changes during TMT, Pearson correlation analyses were conducted.

Statistical analysis was performed using PASW 18.0 software (Tokyo, Japan).

## Results

### Correlation between affective symptoms and QOL

Averaged scores of the SDS, Apathy Scale, and SF-36 are shown in Table 1. As shown in Table 2, the SDS negatively correlated with the SF36-RP (*r* = −0.285, *p* = 0.041), SF36-BP (*r* = −0.279, *p* = 0.045), SF36-GH (*r* = −0.574, *p* < 0.001), SF36-VT (*r* = −0.635, *p* < 0.001), SF36-RE (*r* = −0.434, *p* = 0.002), and SF36-MH (*r* = −0.640, *p* < 0.001). The Apathy Scale negatively correlated with the SF36-PF (*r* = −0.367, *p* = 0.007), SF36-GH (*r* = −0.316, *p* = 0.023), SF36-VT (*r* = −0.459, *p* = 0.001), SF36-RE (*r* = −0.413, *p* = 0.002), and SF36-MH (*r* = −0.433, *p* = 0.001). These results suggest that depressive symptoms and apathy are closely related to a lower QOL.

**Table 1:** Affective symptoms and QOL

	Mean	SD
SDS	36.8	7.7
Apathy Scale	9.8	6.0
SF36-PF	54.9	4.4
SF36-RP	50.5	7.0
SF36-BP	50.6	9.9
SF36-GH	50.9	10.9
SF36-VT	49.0	10.0
SF36-SF	50.7	8.5
SF36-RE	50.3	8.0
SF36-MH	49.4	9.2

*SD* standard deviation, *SDS* Zung Self-rating Depression Scale, *SF36* Medical Outcomes Study Short-Form 36-item questionnaire, *PF* physical functioning, *RP* role functioning, *BP* bodily pain, *GH* general health, *VT* vitality, *SF* social functioning, *RE* role emotional, *MH* mental health

**Table 2:** Correlation coefficients between affective symptoms and QOL

	SDS	Apathy Scale
SF36-PF	−0.261	−0.367**
SF36-RP	−0.285*	−0.273
SF36-BP	−0.279*	−0.207
SF36-GH	−0.574**	−0.316*
SF36-VT	−0.635**	−0.459**
SF36-SF	−0.189	−0.218
SF36-RE	−0.434**	−0.413**
SF36-MH	−0.640**	−0.433**

*SDS* Zung Self-rating Depression Scale, *SF36* Medical Outcomes Study Short-Form 36-item questionnaire, *PF* physical functioning, *RP* role functioning, *BP* bodily pain, *GH* general health, *VT* vitality, *SF* social functioning, *RE* role emotional, *MH* mental health

* *p* < 0.05; ** *p* < 0.01

### Correlation between affective symptoms and task performance

The average TMT time was 75.4 ± 18.3 (mean ± SD) seconds. The score of the SDS was positively correlated with TMT time (*r* = 0.357, *p* = 0.009), suggesting that participants with depression took a longer time to complete the task. In contrast, there was no significant correlation between the score of the Apathy Scale and TMT time (*r* = 0.261, *p* = 0.062).

### Correlation between affective symptoms and [oxy-Hb] changes during task

As shown in Table 3 and Fig. 1, [oxy-Hb] changes during the TMT-A was positively correlated with SDS in CH2 (*r* = 0.442, *p* = 0.021), CH13 (*r* = 0.400, *p* = 0.013), and CH15 (*r* = 0.528, *p* = 0.006) and with Apathy Scale in CH5 (*r* = 0.451, *p* = 0.046), CH13 (*r* = 0.372, *p* = 0.021), CH15 (*r* = 0.0.711, *p* < 0.001), CH22(*r* = 0.339, *p* = 0.017), and CH24(*r* = 0.361, *p* = 0.009). No channel showed [oxy-Hb] changes during the TMT-A that were negatively correlated with the SDS or the Apathy Scale. These results suggest that participants with depression and apathy required greater levels of functional activation in several brain areas to complete the task.

**Table 3:** Correlation coefficients between affective symptoms and [oxy-Hb] changes during TMT

Channels	SDS	Apathy Scale
1	0.38	0.28
2	0.442*	0.36
3	0.28	−0.05
4	0.09	0.09
5	0.18	0.451*
6	−0.09	0.29
7	−0.06	−0.02
8	0.21	0.19
9	−0.01	0.06
10	−0.30	0.17
11	0.11	0.22
12	−0.06	0.21
13	0.400*	0.372*
14	−0.04	−0.12
15	0.528**	0.711**
16	−0.02	0.23
17	0.12	0.22
18	−0.09	−0.01
19	0.06	0.15
20	−0.10	0.08
21	−0.14	0.07
22	0.14	0.339*
23	−0.07	0.16
24	0.06	0.361**

*SDS* Zung Self-rating Depression Scale

* *p* < 0.05; ** *p* < 0.01

## Discussion

In this study, we demonstrated that depressive symptoms and apathy negatively affect brain function and QOL in a non-clinical population. An unexpected, but interesting result was that depressive symptoms had a greater negative impact on task performance than apathy. In this study, we showed that the score of the SDS was positively correlated with the TMT time, but the degree of apathy was not correlated with the TMT time. We also showed that participants with a high degree of depressive symptoms and apathy had a greater [oxy-Hb] increase in many frontal cortical regions.

The degree of depressive symptoms and apathy were associated with most of indices of the SF-36. Our results are consistent with those reported by McCall et al. [17], who showed that an increasing severity of depression was associated consistently with worse QOL in MDD. Our results are also consistent with those of Oguru et al. [21], who reported that both the Apathy Scale and Beck Depression Inventory scores were negatively correlated with QOL in Parkinson’s disease. Together, our results suggest that the presence of depressive symptoms and apathy has a negative impact on individual QOL.

The degree of depressive symptoms was associated significantly with psychomotor slowness, but the degree of apathy was not related to psychomotor slowness. The relationship between psychomotor slowness and age has been shown in previous studies [3, 34]. In our study, age was positively correlated with the TMT time, but there was no correlation between age and affective symptoms (data not shown). Psychomotor slowness in MDD has been shown in previous studies. For example, slower response times in MDD were observed on the TMT, Rule Shift Cards, and Stroop test [6]. Our results are consistent with those reported by Rosenberg et al. [28], who showed that the Geriatric Depression Scale was associated with incident impairment on all cognitive tests including the TMT-A in healthy older women. However, our results are inconsistent with those reported by Feli et al. [4], who showed that apathy correlated with a measure of information processing speed (Stroop test B) in older MDD patients. The reason for this inconsistency is unclear, but one possible reason is a difference between the tasks for psychomotor slowness. The TMT-A may not be sufficiently sensitive to detect the effects of apathy on brain function.

We also showed that participants with high degree of depressive symptoms and apathy had a greater [oxy-Hb] increase in many frontal cortical regions. Previous neuroimaging studies on cognitive impairment in MDD have demonstrated brain activation patterns with hypo-(e.g., Okada et al. [22]) and hyper-(e.g., Walter et al. [38]) activation of frontal cortical regions [13, 37]. In such studies, performance must be taken into account before attempting interpretation, and hyperactivation in context of equal or poorer performance is usually interpreted as ‘inefficiency’. In this study, we found hyperactivation in the context of equal or poorer performance with a high degree of depressive symptoms and apathy, that is, inefficiency. Our results are consistent with those of Wagner et al. [36], who reported prefrontal hyperactivation with equal performance of the Stroop test in MDD using fMRI, and with results of Walter et al. [38], who reported that prefrontal hyperactivation with poor performance of Working Memory task in MDD using fMRI. Our results suggest that participants with a high degree of depressive symptoms and apathy require greater cortical resources to perform the same task. Furthermore, lower QOL and psychomotor slowness caused by depressive symptoms and apathy may be related to such inefficient frontal activation.

Our results are inconsistent with our hypothesis. We found that apathy was associated with low QOL and frontal cortical inefficiency, but was not correlated with psychomotor slowness. One potential explanation is that the effects of apathy may be more sensitively measured by cortical [oxy-Hb] changes detected by NIRS than by behavioral output. Thus, our present methods combining behavioral and NIRS measurement enabled us to detect the effects of apathy on brain function that would be difficult to detect by behavioral output alone.

There are several limitations in this study that should be taken into consideration. First, the participants were all male because women can have potentially influential factor such as mood fluctuations across the menstrual cycle, and our findings may not be generalizable to a female population. Second, assessments of depressive symptoms and apathy are based on self-rating scales without a structured diagnostic interview (e.g., SCID). Third, age and IQ were not controlled. They are potential factors capable of affecting not only psychomotor slowness, but also brain function and QOL. Fourth, depressive symptom was measured using the SDS. Although the SDS was developed specifically for patients with a diagnosis of major depression, the SDS is commonly used even in healthy subject study, since the scale is simple and less burdensome for subjects. Fifth, power analysis and multiple comparisons were not conducted in our study as in most previous NIRS studies. Further studies should take these factors into account. With these limitations in mind, this study provides evidence to support the hypothesis that depressive symptoms and apathy were associated with psychomotor slowness and abnormal cortical activation, as well as low QOL in a non-clinical population.

In conclusion, the degree of depressive symptoms and apathy were associated with lower QOL, and participants with high degree of depressive symptoms and apathy have inefficient cortical activations. On the basis of the findings, we assume that cortical hyperactivation during a psychomotor task measured by NIRS may be used to identify objectively individuals with a high degree of depressive symptoms and apathy. Further functional neuroimaging studies focusing on depressive symptoms and apathy at a non-clinical level may elucidate the brain mechanisms underlying depressive symptoms and apathy. These studies may be beneficial for promoting the QOL of healthy subjects and patients suffering from depressive symptoms and apathy.
